# Resting Brain Activity Related to Dispositional Mindfulness: a PET Study

**DOI:** 10.1007/s12671-017-0677-2

**Published:** 2017-02-09

**Authors:** Martin Gartenschläger, Mathias Schreckenberger, Hans-Georg Buchholz, Iris Reiner, Manfred E. Beutel, Julia Adler, Matthias Michal

**Affiliations:** 1 Department of Nuclear Medicine, University Medical Center of the Johannes Gutenberg-University, Langenbeckstrasse 1, Mainz, D-55131 Germany; 2 Department of Psychosomatic Medicine and Psychotherapy, University Medical Center of the Johannes Gutenberg University, Mainz, Germany

**Keywords:** Mindfulness, Fluorine-18-FDG PET, Default mode, Precuneus, Self

## Abstract

Mindfulness denotes a state of consciousness characterized by receptive attention to and awareness of present events and experiences. As a personality trait, it constitutes the ability to become aware of mental activities such as sensations, images, feelings, and thoughts, and to disengage from judgment, conditioned emotions, and their cognitive processing or automatic inhibition. Default brain activity reflects the stream of consciousness and sense of self at rest. Analysis of brain activity at rest in persons with mindfulness propensity may help to elucidate the neurophysiological basis of this important mental trait. The sample consisted of 32 persons—23 with mental disorders and 9 healthy controls. Dispositional mindfulness (DM) was operationalized by Mindful Attention Awareness Scale (MAAS). Brain activity at rest with eyes closed was assessed by fluorodeoxyglucose positron emission tomography (F-18-FDG PET). After adjustment for depression, anxiety, age and years of education, resting glucose metabolism in superior parietal lobule and left precuneus/Brodmann area (BA) 7 was positively associated with DM. Activity of the left inferior frontal orbital gyrus (BA 47) and bilateral anterior thalamus were inversely associated with DM. DM appears to be associated with increased metabolic activity in some core area of the default mode network (DMN) and areas connected to the DMN, such as BA 7, hosting sense of self functions. Hypometabolism on the other hand was found in some nodes connected to the DMN, such as left inferior frontal orbital gyrus and bilateral thalamus, commonly related to functions of memory retrieval, decision making, or outward attention.

## Introduction

Mindfulness denotes a state of consciousness characterized by receptive attention to and awareness of present events and experiences. Kabat-Zinn (1994, p. 4) described mindfulness as “paying attention in a particular way: on purpose, in the present moment, and nonjudgmentally.” Thus, the ability of mindfulness means the capacity of becoming aware of mental activities such as sensations, images, feelings, needs, and thoughts, without grasping neither into judgments nor strategic planning.

Mindfulness is considered a personality trait (Brown and Ryan 2003) with important implications for mental and somatic health (Kabat-Zinn 1994, Siegel 2007). Traumatic childhood experience may decrease the ability for mindfulness (Michal et al. 2007), while mindfulness-based interventions improve health. Mindfulness-based interventions have shown promising effects on various chronic medical conditions, mental disorders (e.g., major depression, anxiety, somatoform, and personality disorders), as well as on stress reduction in healthy subjects (Chiesa and Serretti 2010, 2011, Heidenreich et al. 2006, Lakhan and Schofield 2013, Sipe and Eisendrath 2012). Improvement of affect regulation by directing attentional resources towards a limbic pathway for present-moment sensory awareness is considered as one of the therapeutic mechanisms of mindfulness exercises (Farb, Anderson, and Segal 2012). In persons without propensity and/or training in mindful awareness, the conscious correlate of brain activity at rest largely consists in mind-wandering with uncontrolled sticking to one or another topic. By contrast, persons with increased mindful awareness are capable to an effortless detachment from particular topics and/or to redirection of their stream of consciousness to a chosen focus. Probably, mindfulness propensity and training in mindful awareness are to be considered as the ability to control mind-wandering and—at least in meditation forms involving a sustained focus of attention—to volitionally redirect consciousness. In such subjects, a modulation of the resting state brain activity is presumed (Vago and Zeidan 2016).

Concerning the neuromorphology of mindfulness, previous studies have shown mindfulness to be associated with specific changes of brain structures and functions (Hölzel et al. 2011, Lazar et al. 2005, Murakami et al. 2012). For example, participation in a mindfulness intervention increased gray matter in the posterior cingulate cortex, the temporo-parietal junction, and the cerebellum as compared with controls (Hölzel et al. 2011). A recent study of default mode connectivity in mindfulness revealed that greater connectivity in the dorsal posterior cingulate cortex and the precuneus predicted dispositional mindfulness in 25 elderly persons (Prakash, De Leon, Klatt, Malarkey, and Patterson 2013). The dorsal posterior cingulate cortex and the precuneus regions are part of the default mode network of the brain, which reflects the stream of consciousness and sense of self during rest (Chiesa, Serretti, and Jakobsen 2013; Whitfield-Gabrieli and Ford 2012, Guo, Kendrick, Yu, Wang, and Feng 2014). The default networks get deactivated as the attentional focus is shifted from the inner world and directed towards the external environment (Whitfield-Gabrieli and Ford 2012). In healthy persons, the default state of the brain includes the medial temporal lobe, the medial prefrontal cortex, and the posterior cingulate cortex, the precuneus, and the medial, lateral, and inferior parietal cortex (Buckner, Andrews-Hanna, and Schacter 2008; Zhang and Li 2012). By contrast, in depression, most studies found reduced lateral prefrontal metabolism and increased medial prefrontal and subgenual cingulate metabolism (Biver et al. 1994; Hosokawa, Momose, and Kasai 2009; Mayberg 1997, 2003).

With these considerations in mind, the aim of the present study was to investigate the characteristics of resting brain activity in relation to dispositional mindfulness. Brain activity at rest with eyes closed was assessed by 2-deoxy-2-(18F) fluoro-D-glucose positron emission tomography (F-18-FDG PET). Regional changes in the utilization of glucose reflect differences in the neuronal activity of the brain. F-18-FDG PET is one of several functional neuroimaging methods. In contrast to functional magnetic resonance imaging (fMRI), the averaged snapshot of the brain state lasts longer (20 min) than that of fMRI. Therefore, correlating brain glucose metabolism with dispositional mindfulness might be a valuable extension of previous studies. Based on the definition of mindfulness and previous findings (e.g., Marchand 2014; Tang, Hölzel, and Posner 2015), we hypothesized that dispositional mindfulness will be associated with a particular pattern of activation and deactivation among components of the default mode network (DMN) and associated brain areas.

## Method

### Participants

The sample consists of 32 right-handed persons (23 patients and 9 healthy persons). The patients had the following diagnoses: major depression *n* = 15, depersonalization disorder *n* = 14, dysthymia *n* = 8, social phobia *n* = 3, generalized anxiety disorder *n* = 4, panic disorder/agoraphobia *n* = 4, obsessive compulsive disorder *n* = 4, posttraumatic stress disorder *n* = 2, 13 persons had a personality disorder (PD), *n* = 8 anxious avoidant, *n* = 1 dependent PD, *n* = 1 paranoid PD, *n* = 1 borderline PD, and *n* = 3 PD not otherwise specified. The healthy controls had no history of any mental disorder and were free of significant symptoms of depression or anxiety (Table 1). Table 1 shows the sociodemographic and clinical characteristics of the sample.

**Table 1:** Sample characteristics stratified for occurrence of mental disorders

	Mentally disordered persons*n* = 23	Healthy persons*n* = 9	Test
Age (years)	30.9 ± 9.0	27.4 ± 5.4	*p* = 0.293
Sex: women % (*n*)	52.2% (12)	55.6% (5)	*p* = 0.863
Years of education (school)	11.5 ± 1.8	13.0 ± 0	*p* = 0.018
Depression (BDI-II)	28.1 ± 14.4	1.8 ± 1.7	*p* < 0.001
Anxiety (STAI-T)	59.4 ± 11.5	31.2 ± 5.4	*p* < 0.001
MAAS	3.3 ± 1.0	4.9 ± 0.6	*p* < 0.001

Data are given as mean ± standard deviation with exception of sex (percentage %, numbers). *BDI-II* Beck Depression Inventory-II, *STAI* State-Trait Anxiety Inventory, *MAAS* Mindful Attention Awareness Scale; Test, *t* test for continuous variables and Chi-square test for categorical variables

Persons with a lifetime diagnosis of a psychotic disorder, brain damage, or current medical diseases were not eligible. Each participant gave his written informed consent prior to study participation. Patients were recruited from the Department of Psychosomatic Medicine and Psychotherapy (Mainz, Germany); healthy controls were recruited by research advertisement. Exclusion criteria were all kind of physical diseases or general poor physical health status interfering with the parameters under examination or with the completion of the study protocol, pregnancy and breast feeding, incapacity to follow the study protocol, limited or missing legal competence, and acute suicidality. All participants were in the normal range in routine laboratory tests (blood cell count, blood glucose, electrolytes, γ-GT, creatinine, C-reactive protein) and blood pressure measurement. All participants were paid an allowance of €100 (≈ $130) for their participation.

### Procedure

Before injection of the tracer, the participants were for 30 min in the standardized resting condition, lying with an eye mask quiescently in a supine position. The tracer consisted of 180 MBq F-18-FDG with an estimated effective dose of 3.4 mSv. After injection of the tracer, the participants laid for another 20 min in the standardized resting condition. Afterwards, all scans were acquired in 3D mode by a Siemens ECAT Exact scanner (Siemens, Knoxville, USA). The scanner offers an axial field of view of 16.2 cm and an axial resolution of ~6.0 mm FWHM. A sequence of three 5-min frames was obtained and later combined into a single frame. After correction for attenuation, scatter, and dead time, images were reconstructed by filtered back projection using a 4-mm Hamming filter. Images were resliced in the AC-PC orientation.

### Measures

Dispositional mindfulness was measured with the German version of the Mindful Attention Awareness Scale (MAAS, Brown and Ryan 2003, Michalak, Heidenreich, Ströhle, and Nachtigall 2008). The MAAS consists of 15 items addressing the general tendency to be attentive to and aware of one’s experiences in daily life. The items describe *day-to-day* experiences such as acting as if on automatic pilot, or not paying attention to the present moment (e.g., “I rush through activities without being really attentive to them”). Items are endorsed on a 6-point Likert-type scale, ranging from almost always (=1) to almost never (=6). Scores may range from 1 to 6, with high scores reflecting high dispositional mindfulness. The MAAS is considered as a valid and reliable scale of mindfulness. The MAAS was able to separate experiences meditators from a community sample (Brown and Ryan 2003).

As potential confounders and for the description of mental distress, the Beck Depression Inventory-II (BDI-II; Beck, Steer, Ball, and Ranieri 1996) and the trait version of the State-Trait Anxiety Inventory (STAI) (Spielberger, Gorsuch, and Lushene 1970) were applied. The BDI-II measures severity of current depression, the STAI for trait anxiety.

### Data Analyses

Data were presented as numbers and percentages (%) or mean ± standard deviation. Sociodemographic and psychometric characteristics were compared by *t* test or Chi-square test. Normal distribution of the MAAS was tested by the Kolmogorov-Smirnov test. Bivariate correlation Pearson coefficients were calculated for the psychometric scores. These calculations were done with SPSS 20.

For imaging, data spatial normalization was applied. Voxelwise statistics were calculated using SPM 8 (Wellcome Trust, London, G. B.) and MathLab 7.9 (Mathworks, Natick, MA, USA) in the form of two-sample *t* tests. Significant clusters of activation were determined using height and extent thresholds of *p* < .05 corrected for multiple comparisons using SPM’s small volume correction on cluster level. In order to increase statistical validity, we did not consider any clusters containing fewer than 20 voxels. Normal distribution was tested using the Shapiro-Wilk Normality test. As effect size estimates, we report Cohen’s *d* (z-max). Multiple regression analysis was obtained by means of SPM 8. Location of the activations was accomplished by use of the Montreal Neurological Institute (MNI) standard brain. Associations of brain glucose metabolism with the subjective ratings were examined by linear multi-regression-analysis. The dependent variable was dispositional mindfulness as measured by MAAS. The regression coefficients were corrected for age, sex, years of education, depression (BDI-II), and anxiety (STAI).

## Results

MAAS scores were normally distributed within the sample (Kolmogorov-Smirnov-*Z* = 0.626, *p* = 0.828). The MAAS mean score of the sample was 3.78 and the standard deviation was 1.15. The lowest MAAS score was 1.33 and the maximum score was 5.93. Reliability of the MAAS proved to be excellent (Cronbach’s alpha = 0.932). MAAS correlated negatively with depression (BDI-II, *r* = −0.55, *p* = 0.001) and anxiety (STAI, *r* = −0.72, *p* < 0,001), positively with age (*r* = 0.36, *p* = 0.041) but not with years of education (*r* = 0.075, *p* = 0.683).

**Fig. 1:**
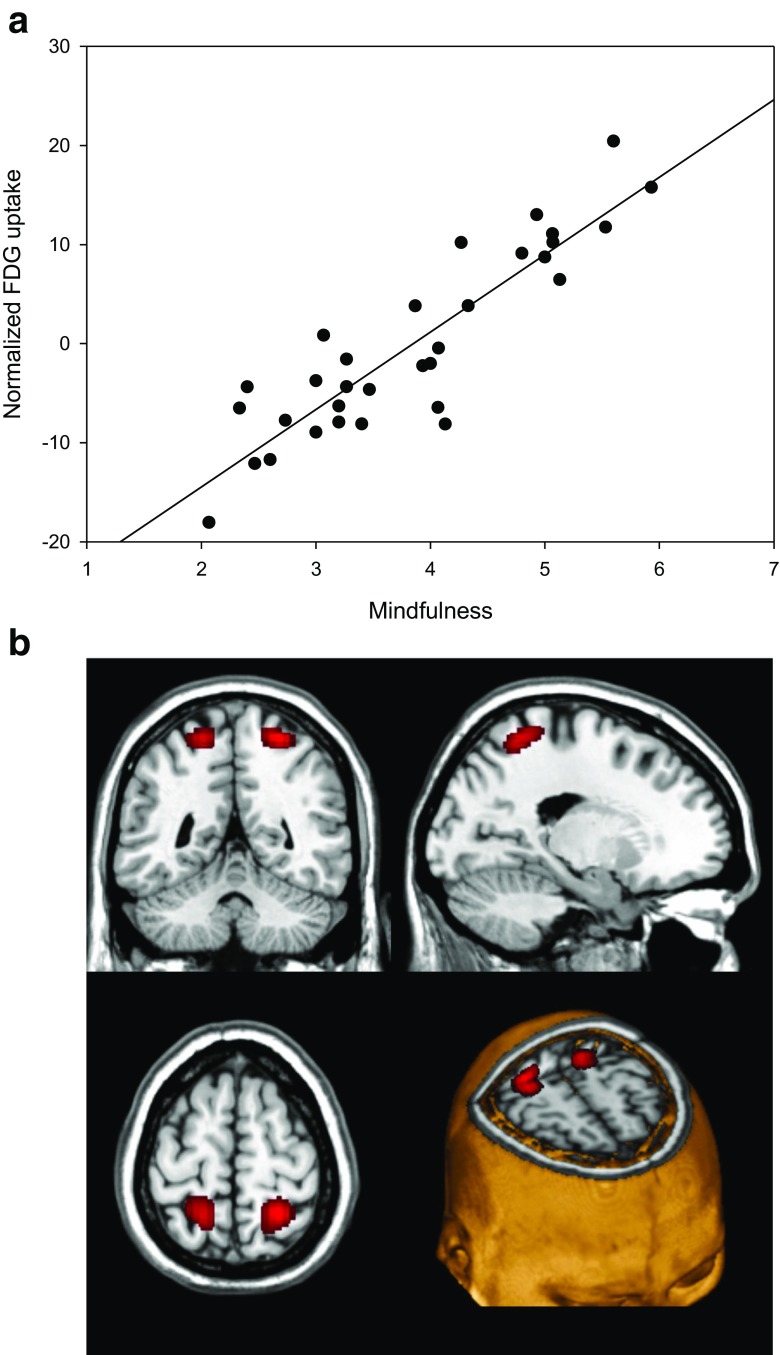
Positive correlation between maximum regional glucose metabolism in Brodmann area 7 and dispositional mindfulness as assessed by MAAS, *p* < 0.001 uncorrected (corrected *p* = 0.002 at cluster level after small volume correction (SVC)). **a** Scatterplot with regression line (*r* = 0.89). **b** Overlay statistical map (threshold *p* < 0.001 uncorrected) on standard MRI

**Fig. 2:**
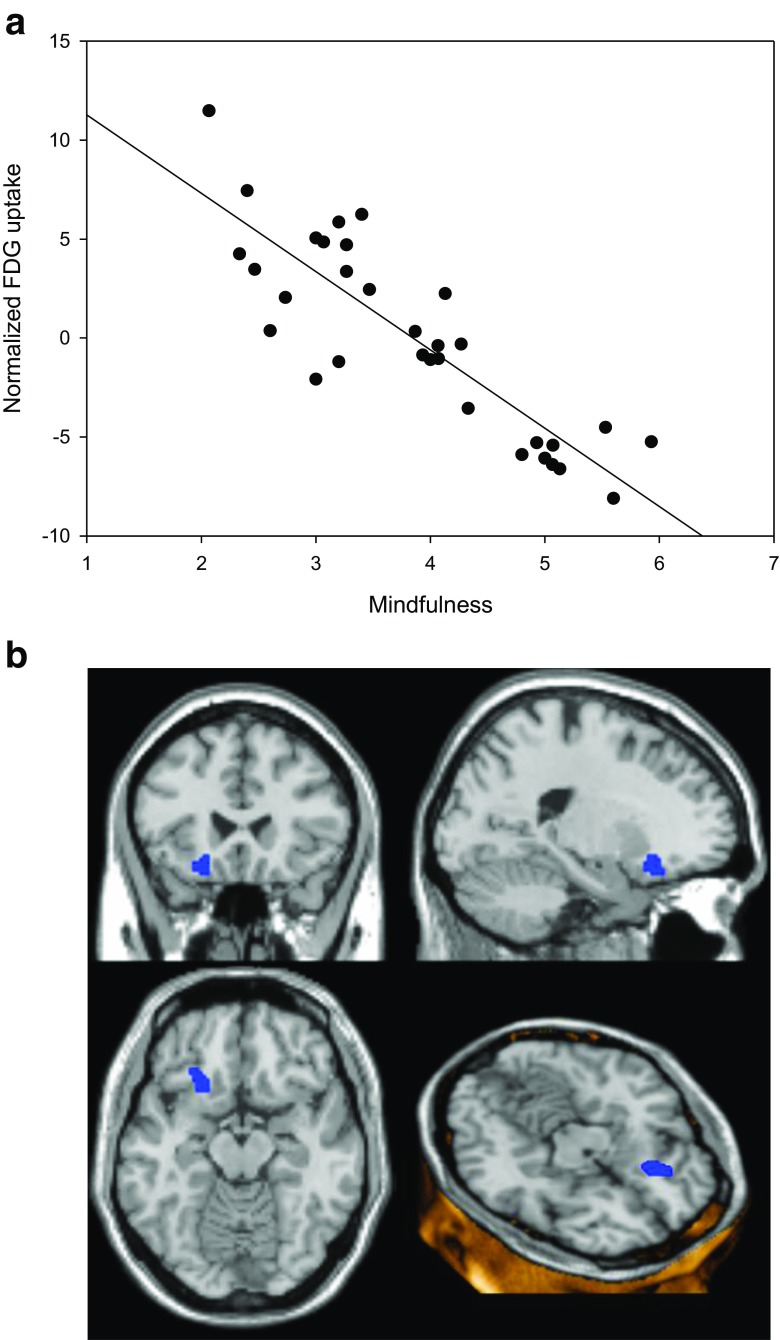
Negative correlation between maximum regional glucose metabolism in Brodmann area 47 and dispositional mindfulness as assessed by MAAS, *p* < 0,001 uncorrected (SVC-corrected *p* = 0.005 at cluster level). **a** Scatterplot with regression line (*r* = 0.86). **b** Overlay statistical map (threshold *p* < 0.001 uncorrected) on standard MRI

**Fig. 3:**
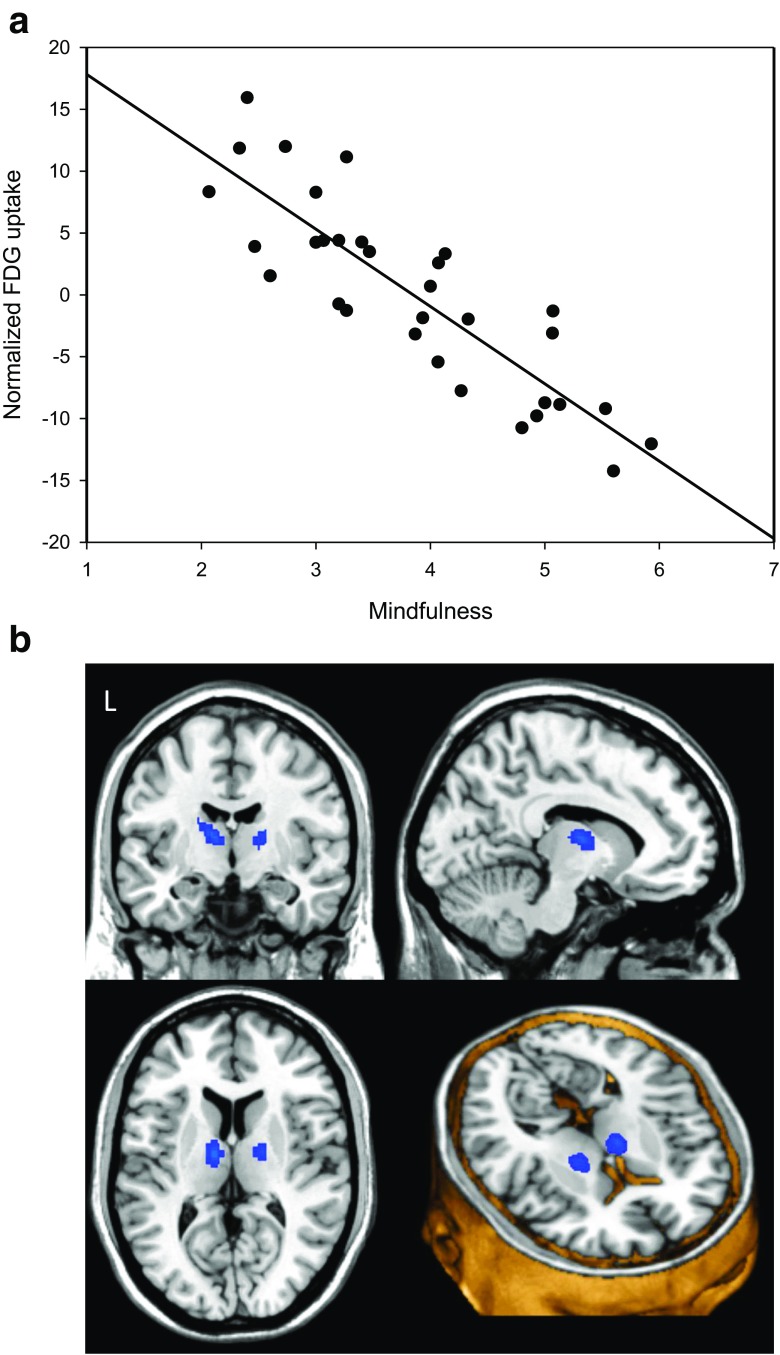
Negative correlation between maximum regional glucose metabolism in the anterior thalamus and dispositional mindfulness as assessed by MAAS, *p* < 0.001 uncorrected (SVC-corrected *p* = 0.004 at cluster level). **a** Scatterplot with regression line (*r* = 0.86). **b** Overlay statistical map (threshold *p* < 0.001 uncorrected) on standard MRI

To demonstrate associations between dispositional mindfulness and regional glucose metabolism, we entered trait levels of mindfulness disposition in our higher-level model, after co-varying out depression, anxiety, age, and years of education. Resting glucose metabolism correlated positively with dispositional mindfulness in right superior parietal lobule, within Brodmann area (BA) 7 and to a minor extent BA 5 and left precuneus and superior parietal lobule, in a region overlapping BA 7 > 5 and minimally BA 40, as illustrated in Fig. 1. By contrast, metabolic activity in the left inferior frontal orbital gyrus (BA 47) and in bilateral anterior thalamus correlated inversely with dispositional mindfulness, compare Figs. 2 and 3. Details of the relationship between local energy consumption and dispositional mindfulness are given in Table 2.

**Table 2:** Correlation of brain metabolic activity with dispositional mindfulness

	Side	Coordinates [x, y, z]	*Z*-score	Cluster size
Positive correlation with dispositional mindfulness (MAAS)				
Precuneus and superior parietal lobule (BA 7 > 5)	L	−18, −50, 64	3.74	374
Superior parietal lobule (BA 7 > 5 > 40)	R	26, −50, 64	3.98	356
Negative correlation with dispositional mindfulness (MAAS)				
Inferior frontal orbital gyrus (BA 47)	L	−24, 26, −18	3.54	83
Anterior thalamus	R	18, −8, 10	3.43	31
Anterior thalamus	L	−10, −10, 10	3.55	87

At *p* < 0.05 corrected for multiple comparisons on cluster level

## Discussion

Dispositional mindfulness was positively associated with resting glucose metabolism in the right superior parietal lobule within Brodmann areas 5/7 and in left precuneus and superior parietal lobule and negatively with the metabolic activity of the left inferior frontal orbital gyrus (BA 47) and bilateral anterior thalamus. Brodmann area 7 constitutes a core structure of the default brain network. Its activity reflects multisensory integration, complex representations of the body (Felician et al. 2004; Fortin, Ptito, Faubert, and Ptito 2002), and the representation of the mental self (Cavanna and Trimble 2006; Lou et al. 2004). Data from the literature supports the implication of superior parietal lobule in meditative and spiritual activity (Beauregard, Courtemanche, and Paquette 2009). The inverse association of dispositional mindfulness with bilateral thalamus activation may be interpreted as reflecting a state of reduced arousal and reduced outward directed attention (Fan, McCandliss, Fossella, Flombaum, and Posner 2005; Huber, Lui, and Porro 2013). This finding is in line with Wang et al. (2014), who found in a fMRI study on the DMN a low involvement of the thalamus correlating with high levels of trait mindfulness (Wang et al. 2014). They suggested the concept of the thalamus as a switch between mind-wandering and mindfulness.

There is experimental evidence for the anterior nucleus of the thalamus (ANT) constituting a pivotal node in the limbic circuitry which supports fear conditioning (Marchand, Faugère, Coutureau, and Wolff 2014). Negative correlation between ANT metabolism and mindfulness trait may represent the functional substrate of an increased ability to disengage from conditioned emotional charges.

Further, activity of the left inferior frontal orbital gyrus (BA 47) correlated negatively with trait mindfulness. BA 47 has been found to be associated with language-related functions (Belyk and Brown 2014), emotional control and inhibition (Beauregard 2007; Beer et al. 2006; Berthoz, Armony, Blair, and Dolan 2002; Phillips et al. 2001), decision making (Rogers et al. 1999) and deductive reasoning (Goel, Gold, Kapur, and Houle 1997, 1998), controlled memory retrieval, and— jointly with core structures of the DMN—creativity tasks (Beaty et al. 2014). A recent study on episodic source memory retrieval revealed strong coactivation of precuneus and BA 47. In this context, activity of the left inferior prefrontal/frontal operculum (BA 47) was interpreted as reflecting the attempt to integrate retrieved information (Lundstrom, Ingvar, and Petersson 2005). Concerning the role of BA 47 for emotional processing, previous findings supposed that BA 47 activity processes the automatic regulation of emotional behavior (Phillips, Drevets, Rauch, and Lane 2003). Interestingly, abnormal activity of BA 47 has been found in patients with depersonalization disorder in response to emotional stimuli (Phillips et al. 2001). The authors concluded that increased activation of the right BA 47 is responsible for inhibition of emotional experiencing in patients with depersonalization disorder (Phillips et al. 2003). This finding may be of interest for this analysis, as depersonalization may be considered the antithesis of a mindful state (Allen 2005, Nestler, Sierra, Jay, and David 2015, Michal et al. 2007).

Taken together, our findings are in part in line with recent studies of default-network connectivity, which demonstrated increased connectivity of the DMN, specifically in the precuneus and the dorsal posterior cingulate cortex with mindfulness disposition (Prakash et al. 2013, Marchand 2014). Differences of our study may be due to diverse technical approaches, experimental design (resting state versus meditating; type of instruments for determining mindfulness), and sample size and characteristics. As Tang et al. (2015) discussed recently in their review, despite numerous neuroimaging studies of mindfulness meditation and overlapping results (e.g., Marchand 2014, Tang et al. 2015), the precise underlying neural mechanisms remain still unclear. In sum, our findings may suggest that at rest, dispositional mindfulness correlates positively with BA 7, area responsible for attending to the sense of self and inversely with BA 47, reflecting lower activity of language processing and automatic emotion regulation (Phillips et al. 2003) and with bilateral thalamus reflecting a state of low arousal (Fan et al. 2005; Huber et al. 2013).

While making the above considerations, several limitations have to be kept in mind. Although a standard measure of mindfulness, MAAS scores may be only an approximate measure of mindfulness. Important to note, we measured glucose uptake after 30 min resting in a supine position with eyes closed, not during a standardized mindfulness exercise task. Thus, the design of our study does not include the aspect of paying attention *on purpose*. This is to say that our results preclude any conclusions about the brain activity during actual mindfulness exercises. It is important to bear in mind that we related habitual brain activity during resting state with a trait measure of mindfulness. Further, due to our sample composition, we cannot preclude that psychopathology may have confounded our results. However, we were able to describe severity of depression and anxiety in the sample and to adjust for these variables, which should reduce their potential confounding effects. A further limitation is that both samples differed regarding education. However, to correct our analysis for this imbalance, we adjusted for years of education.

In conclusion, our study on the resting brain activity of dispositional mindfulness revealed strong activations of BA 7 areas (precuneus/superior parietal cortex) reflecting increased attendance to the sense of self and diminished activity in the following two brain areas: (a) BA 47, presumably reflecting less semantic processing, less memory retrieval, less evaluation of contextual relevance of emotional information, less decision making, and automatic emotion inhibition, and (b) bilateral thalamus, reflecting a state of low alertness, reduced outward attention and disengagement from conditioned emotions, and possibly from mind-wandering. Therefore, dispositional mindfulness may be considered as a trait towards increased attention to the current sense of self and an increased ability to disengage from conditioned emotions and from their cognitive processing or automatic inhibition.
